# Stakeholders’ Perspectives on the Quality of End-of-Life Health Care Services for Chronic Obstructive Airways Disease: A Focus Group Study

**DOI:** 10.5334/ijic.7274

**Published:** 2023-08-08

**Authors:** Amanda Landers, Suzanne G. Pitama, Suetonia C. Palmer, Lutz Beckert

**Affiliations:** 1 Department of Medicine, University of Otago, Christchurch, New Zealand; 2 University of Otago, Christchurch, New Zealand

**Keywords:** chronic obstructive pulmonary disease, healthcare network, quality healthcare, healthcare systems, human factors

## Abstract

**Introduction:**

Delivery of end-of-life care for severe chronic obstructive pulmonary disease (COPD) has been hampered by an unpredictable disease trajectory and poor integration of health care and social services.

**Objective:**

To critically explore the perspectives, values, and experiences of stakeholders in COPD end-of-life healthcare services in a large district in Aotearoa New Zealand.

**Design:**

Focus groups analysed utilising critical theory and Actor-Network Theory.

**Methods:**

Stakeholders in end-of-life COPD healthcare services were purposively sampled from a large healthcare network in Canterbury, Aotearoa New Zealand to participate in seven focus groups (bereaved carers, community-based health professionals, non-Māori, non-Pacific patients, and support people (two groups), Māori patients, supporters and health professionals, Pacific patients, support people and health professionals, and hospital-based health professionals). Participants discussed end-of-life care services for people with COPD. Transcripts were coded utilising descriptive and structural coding to develop themes related to provision of quality care. Participants were positioned as experts. We considered how the themes arising supported and disrupted the healthcare network for end-of-life COPD.

**Results:**

Five themes related to quality of care for end-of-life COPD were identified: compassion, competence, community, commitment, and collaboration. The absence of any of these five themes required for quality care led to power imbalances within healthcare systems. Power inequities created disconnection among stakeholders which then disrupted commitment, community, and collaboration. A dysfunctional healthcare network impeded compassion between stakeholders and did not support their competence, leading to lower quality care. All five themes were identified as essential to delivery of high-quality end-of-life care in COPD.

**Conclusion:**

Stakeholders’ perspectives of end-of-life care for COPD identified of core features of a health system network that enabled or impeded the actions of stakeholders and allocation of resources to provide quality care.

## (1) Introduction

Chronic obstructive pulmonary disease is the third-leading cause of death globally [1]. COPD is a debilitating lung condition that causes progressive disability and breathlessness, in an unpredictable trajectory [2]. In Aotearoa New Zealand (NZ), the prevalence of COPD is declining, currently affecting 2.2% of the population [3]. However, the hospitalised prevalence is higher for Māori (2.5%) and Pacific (2.44%) Peoples [3]. The socio-economic and ethnic differences in hospitalisations due to COPD has widened between 2000 and 2019 [3] while end-of-life care for people with COPD is poorly integrated [4]. Health professionals in Aotearoa New Zealand have identified challenges in the provision of care for this population, specifically in availability, suitability and affordability of services [5]. Healthcare systems providing end-of-life care for COPD are rarely informed by patients, their carers or health professionals, who lack influence on system design and resourcing [4,6].

Toward the end-of-life, people with COPD experience multiple hospital admissions, social isolation, and increased need for health services [2,7]. Traditionally, end-of-life care programmes have aligned their structure with cancer diagnoses which are characterised by clinical predictability and rapid health deterioration, distinctly marking end-of-life [8]. In cancer care, choosing not to pursue disease-modifying treatments marks the turning-point towards end-of-life goals. No such transition point exists for COPD and few patients receive dedicated palliative-focused management [8]. This lack of specific signal for end-of-life can lead to an increasing number of stakeholders involved in care, with differing roles and responsibilities and increasing risks of service disintegration.

Early integration of palliative care services improve symptom burden, referral to timely care and the rates of advance care plan completion [9,10]. People with COPD who did not receive end-of-life care services in the last three months of their lives more frequently died in acute care settings [11]. Those who received community-based end-of-life care services experienced less days in hospitals [11]. The elements of these end-of-life services that influences high quality of the care is less obvious in the literature.

The NZ Ministry of Health describes the need for a systematic management approach to long-term conditions, which includes patient-centred and proactive care, achieving equity of outcomes and a resource-sustainable system [12]. This government approach of integration and personalisation is found in other jurisdictions [13,14]. Healthcare in Aotearoa New Zealand is a mixture of public and private organisations, with subsidised services for children and long-term illnesses. Primary care and community services have key roles in delivering services to patients with long-term conditions such as COPD. However, the health system has poorly integrated services and funding [15]. This phenomenon led the research team to consider how stakeholders’ experiences of delivering or receiving care demonstrates how the health network for end-of-life COPD is supported and disrupted as a marker of quality of care. Healthcare networks describe the relationships between individuals, organisations and groups involved in the delivery of health.

The aim of this paper was to critically explore the experiences and perspectives of stakeholders of the healthcare system for end-of-life COPD to elucidate how the healthcare network enables or disrupts high-quality care.

## (2) Methods

The research team conducted a qualitative study utilising seven focus groups to position people with severe COPD, their support people, and clinicians as experts in the evaluation of end-of-life health care services. Focus groups were chosen to facilitate collective discussion of a series of questions about the model of healthcare experienced by participants. The research team chose the Canterbury region as Aotearoa New Zealand’s second-largest urban area. The number of people living in Canterbury is approximately 600,000 which represents 12.7% of Aotearoa New Zealand’s population [16]. People identifying as Māori and Pasifika represent 9.4% and 3.2% of the population, respectively.

We utilised Critical Theory and Actor-Network Theory (ANT) as the frameworks to interpret the data, centring stakeholders’ voices. Critical Theory explores the power in a system, identifies who holds that power, and analyses the impacts of the positioning of power in observed phenomena [17]. In our research, this theory was employed to critique care quality based on the socio-political contexts of participants within health care services that distribute power. Actor Network Theory investigates the interactions within a system, including inanimate processes, resources, objects and ideas, as well as human activity, to describe and explain social phenomena [18]. ANT enabled us to describe stakeholder experiences nonhierarchically within the healthcare system inclusive of people and resource considerations.

The focus groups included patients with severe COPD, their support people and health professionals across community and hospital settings. Patients and their support people were included within the same focus groups. Health care providers included general practitioners, district nurses, clinical nurse specialists, allied health and specialist teams. Specific Māori and Pacific focus groups were co-facilitated and led alongside Māori and Pacific health researchers to ensure cultural safety. A bereaved support people focus group also formed due to a request from the community.

**Figure 1:**
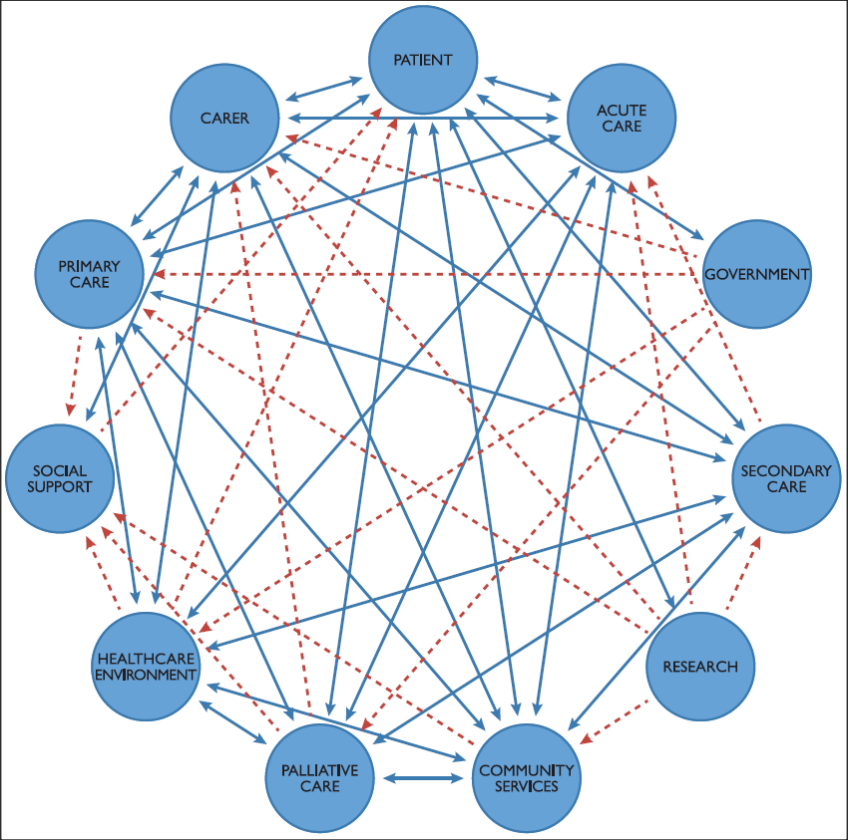
The Health Service Network for Severe COPD- a model of care.

The interview schedule explored the experience of end-of-life care services for people with severe COPD. During the orientation of participants at each focus group, we shared a network diagram of the stakeholders involved in care delivery derived from a systematic review of qualitative studies [4] (Figure 1).

This diagram represents the 11 stakeholder groups (blue circles) and their interactions with other stakeholders in the network. The solid blue arrows indicate two-way interactions between groups and the dotted red arrows represent one-way interaction [4].

Participants were then invited to describe encounters with stakeholders during their care and how they perceived integration of their care within the system.

All the transcripts were transcribed and read several times by the first author, AL. AL presented preliminary data to all the investigators for discussion at each step of the analysis. Coding involved all investigators, where the criteria for coding patterns and defining of inclusion and exclusion criteria for codes, categories and themes were discussed, negotiated, and agreed. The first cycle of analysis involved deductive thematic analysis through structural coding to organise the data, and then descriptive coding using a deductive approach to develop codes within the data [19]. The second cycle of analysis involved two iterations of pattern coding to identify relationships between codes, to form categories, and then to organise categories into themes [19].

## (3) Results

Seventy-four participants were included in seven focus groups (Table 1).

**Table 1:** Characteristics of participants across the seven focus groups n = 74.


FOCUS GROUP	PARTICIPANTS	PATIENTS	SUPPORT PEOPLE	HEALTH PROFESSIONALS

1	Bereaved carers		5	

2	Community-based health professionals			14 Nursing n = 7 Medical n = 4 Allied Health n = 3

3	Non-Māori, non-Pacific patients and support people	8	3	0

4	Non-Māori, non-Pacific patients and support people	10	2	0

5	Māori patients and support people and health professionals	5	1	1

6	Pacific patients and support people and health professionals	1	3	5

7	Hospital-based health professionals			16 Nursing n = 4 Medical n = 8 Allied Health n = 4


Five themes related to the quality of end-of-life care within a healthcare network were identified: compassion, commitment, competence, collaboration, and community (Table 2).

**Table 2:** Care Model Themes, their definitions and their influence on the healthcare network.


THEME	DEFINITION	STRENGTHEN THE NETWORK	DISRUPT THE NETWORK

Compassion	Compassion is aware of and responds to the suffering and distress of others	compassion listens (aware of) compassion acts beyond the usual (responds to) compassion understands (aware of) compassion cares deeply for others (responds to) compassion takes away burdens (responds to) compassion supports (responds to) compassion is interested (aware of)	compassion sometimes breaks the rules (responds to) lack of compassion demoralises (aware of and responds to) lack of compassion undermines trust (responds to) lack of compassion shames (responds to) lack of compassion creates anger (responds to)

Commitment	Commitment is dedication to and engagement with another person or organisation	commitment makes promises (dedication and engagement) commitment provides continuity of care (dedication) commitment fulfils duty (dedication and engagement) commitment is persistent (dedication) commitment leads to expectations being exceeded (dedication and engagement) commitment is part of loving someone (engagement) commitment is being reliable (dedication) commitment is having courage (engagement) commitment is consistent (dedication)	commitment makes promises that sometimes cannot be kept (dedication and engagement) lack of commitment stops continuity of care (dedication) commitment can feel like a duty (Dedication and engagement) lack of commitment lets people down (dedication) lack of commitment leads to inconsistencies (dedication)

Competence	Competence is knowledgeable and proficient, with good communication skills	competence knows about end-stage COPD (knowledgeable) competence is pro-active (proficient) competence is efficient (proficient) competence communicates the treatment and plan (communication) competence is accurate (knowledgeable and proficient) competence knows what to do in a crisis (knowledgeable and proficient) competences assess and reviews (proficient) competence is up to date (knowledgeable) competence is culturally aware (knowledgeable)	lack of competence in the management of end-stage COPD (knowledgeable) lack of competence creates gaps in knowledge (communication) lack of competence creates gaps in care (knowledgeable and proficient) lack of cultural competence creates inequities (knowledgeable)

Collaboration	Collaboration is a team player and shares responsibility for decisions around healthcare	collaboration gives seamless care (team and responsible) collaboration is flexible (team) collaboration likes to find solutions (responsible) collaboration opens doors (team) collaboration knows own limitations (team) collaboration shares control (team and responsible) collaboration is aware and respectful of other team players (team) collaboration validates everyone’s experience (team) collaboration encourages others to participate	lack of collaboration removes choices from team members (responsible) lack of collaboration impacts knowledge transfer (team) lack of collaboration invalidates peoples’ experiences (team and responsible) lack of collaboration disconnects team members (team)

Community	Community shares common interests with others and experiences a sense of belonging	community understands each other’s stories (common interests) community puts others first (belonging) community supports family culture (belonging) community creates social interaction (belonging) community gives purpose (belonging) community has knowledge of the illness (common interests) community crosses boundaries and is inclusive (belonging) community steps into the gaps (belonging)	lack of community can lead to lack of advocacy (common interests) lack of community creates an ‘us’ and ‘them’ (belonging) lack of community causes isolation and loneliness (belonging) lack of collaboration leads to exclusion and judgement (belonging)


These themes will be further explained within the context of how analysis indicated they strengthened or disrupted the network for end-of-life services for people with severe COPD.

### (3.1) Compassion

Compassion included the participants’ signposting the awareness of, and response to, the suffering and distress of others. Patients and their support people consistently described compassion from all health care providers as a feature of quality care for end-of-life COPD.

Participants reported interactions in the healthcare system when a stakeholder displayed compassion. These encounters inspired trust and improved quality of life.

*They were fantastic over there. They don’t push you. They don’t force you. […] It’s his home and it’s your home. You can sleep there if you want. You come whenever you want. The kids were there. […] We had all the memorabilia on the wall. (Focus Group 1, Support person)*

The participants described compassionate organisations as those with health care workers that listened, and were supportive, interested, flexible and understanding. Health professionals who worked in community-based services recognised the part they played in reducing isolation and loneliness.

*A lot of our district nursing visits for palliative care is five minutes’ help and the rest is they just want to talk, talk to someone about their day. A lot of the people we see we might be the only person they see that day, if not that week. But there is a lot of isolation and lack of mobility (Focus Group 2, Community health professional)*

Patient and support people described acts of compassion as meaningful to them. These actions included asking after a person’s health, remembering their name, or asking if they needed anything. Participants described feeling connected and having faith in members of the health care team who were kind, in ways that the participants’ considered might have seemed trivial to the health care professional.

*I’ve got the best doctor in the world… […]. Nobody can get in to see her now. If you want something done, she’s on the phone straight away. And my husband went in the other day to pick up something for me and she insisted that the receptionist say when he’s there […] and she said oh how is […]? How is she you know? Is there anything she needs? (Focus Group 3, Patient)*

Several patients and support people reported encounters with the public, government departments, and health professionals that lacked compassion. These encounters included: not being believed over important details of their own personal circumstances, lack of respect, and abusive interactions.

*I even got told off by a woman the other day for using the disability toilet. […] this woman was there. I know her quite well. She is on a walking frame and that, she’s got cerebral palsy or something. Anyway, she said, “What the hell do you think you are doing using that toilet? I said I’m having a pee like you’re going to.” […] And she said, “You’ve no right to use that toilet.” I said, “Why?” and she said, “It’s only for disabled people. (Focus Group 4, Patient)”*

The lack of compassion they encountered in various scenarios perpetuated and heightened the power imbalances between patients, and their health professionals, friends, family, and community. These interactions caused patients to feel demoralised and ashamed. This led patients to feel unworthy and undeserving of services and help. They often linked this with the stigma of perceptions that they had caused their illness through smoking. These power imbalances caused disconnection with, and avoidance of, services that may have had therapeutic benefit for patients and their support people.

### (3.2) Commitment

Commitment was discussed by participants as those dedicated to and engaged with the health care of the patient, support people or collegial relationships between health professionals. Focus group respondents reported the importance of commitment to attain consistent and reliable health care.

Support people and health professionals described their commitment to people with (end-of-life) COPD as the driving force behind their advocacy. Their voices were needed to ensure that all services were delivered in an effective and timely way.

*I think a 100% with my dealing with my brother, I was my brother’s voice. I’m pretty outspoken. If I think someone needs something, I’ll do it and I’ll go that extra mile 100% and I’ll fight for that and my brother… […] I had to learn what his saturation levels had to be and it couldn’t be below or higher but it’s been the voice for them- it is a great deal to know. When you go into the ED, they are asking him things and I am like-… he can’t breathe, he can’t talk. You can’t ask any stuff like that; you need to ask me. […] I was his voice. I was his advocate for Work and Income, everything. Every possible thing, I was his voice. (Focus Group 1, Support person)*

Support people described how they took on progressively more care over time including organising and administering medications and equipment, providing meals and food, and ultimately moving in to live with the patient. These participants described carrying out intimate care for their relatives which was not a task they originally expected to be performing. Support people took on these roles as a commitment to their relative or friend and discovered courage through fulfilling their duty. Patient and support people viewed the willingness of health providers to negotiate and work with families beyond what they expected as commitment. This was demonstrated by being understanding, reliable, consistent, available, and responsive to needs. Participants also described commitment to facilitating a good death as high-quality care.

*They (aged residential care facility) were so supportive and really hands on with their care and they understood. So, the BiPAP was there, which he was on BiPAP for a long time anyway and they were amazing to find somewhere that understood his breathing. Didn’t force him to do stuff, didn’t, they checked on him and that was his biggest fear of dying alone even though the carers were there to do everything for him. (Focus Group 1, Support person)*

The progressive increase in caring responsibilities caused enormous stress for support people even while they were strongly committed to their relative or friend. These participants described the loss of their relationship as a wife, partner, or friend as they became carer. As well as personal care, support people made critical decisions on behalf of their relatives and themselves, describing this as being to their own detriment. These decisions were frequently as described emotional and life-changing for all concerned.

*I lay in bed and bawled and bawled for two nights and then I thought, ‘I’m going to look round these rest homes. I told no one. I didn’t want anyone with me. […] It just about exhausted me going round all these different places. (Focus Group 1, Support person)*

Patients described a medical culture that lacked commitment to learning about the importance of support groups and the function they play in the health system for advanced COPD. Participants were disappointed by a lack of commitment by the health system to shifting towards integrating social services. They perceived a political divide and power imbalance between their support systems, and the medical profession. Individuals and organisations were only willing to commit to patients and their support people in certain ways, but not always the ways that they described as being the most helpful.

*sometimes they go to the doctors to take the Windmill, which is our Better Breathing little magazine if you want to call it that, […] and the first person you meet of course is the receptionist. I don’t want her to just plant it inside on the walls like here, […] I want the doctors to read it, […] and to understand please send them there or ours. (Focus group 5, Māori patient)*

### (3.3) Competence

Competence was drawn from descriptions by participants of being knowledgeable and proficient, with good communication skills. Competence as a component of high-quality care was discussed in all the focus groups.

Patient and support people strongly valued health professionals who were knowledgeable about severe COPD. Knowledge and competencies with up-to-date medication prescribing and inhaler management was particularly important to patients and support people.

*I was on two inhalers, and she (pharmacist) put me onto four, changed everything really, and it just gave me a whole new life basically. I could do things that I couldn’t do before. I didn’t realise that they were trained for that, the special job that they do. And that was great. (Focus group 4, Patient)*

There was a strong thread of discussion around health care workers who appeared to understand the illness, have knowledge that was helpful to all involved, and acted on it quickly and efficiently. Participants trusted health professionals who were pro-active when managing end-of-life COPD, such as arranging hospital admissions for antibiotics, organising home alterations, and assisting with the transition to residential care. Participants described encountering a lack of knowledge and proficiency amongst members of their health care team. This included a lack of understanding about oxygen use, checking they have the correct patient, commencing the correct medication and knowing how it works.

*Yeah, but I don’t think they understand COPD. If I ring the bell for something they’re good like that, but I don’t think they understand the disease. (Focus Group 4, Patient)*

Competence also encompassed good communication skills from health care professionals to the patients and their support people. They described the provision of information and education about their treatment plans as reflective of high-quality care. Inadequate communication skills by health professionals with patients and their support people lead to uncertainty about the health care plan, furthering power imbalances between those utilising health services and those providing it. This was particularly evident as a discussion among participants in the Māori and Pacific focus groups, with the added importance of health professionals needing to be culturally safe.

*I felt like there were some good nurses who we dealt with but there were those who weren’t culturally competent enough to deal with dad so yeah it was, I struggled with palliative care at one stage […] it was like they just came in, did their job you know, put the pump, put the morphine in and […] there was nothing about listening to what dad was going through (Focus group 6, Pacific support person)*

Māori and Pacific participants discussed the lack of understanding of their cultural worldview among non-Māori, non-Pacific health professionals. Patient and support people reported encounters with multiple health professionals that did not engage or educate whānau (family and kin). The health system appeared to focus on physical health, disregarding the social, emotional, and spiritual impact of ill-health. Pacific participants discussed the lack of acknowledgement of diversity within the Pacific region by health professionals. This led participants to consider that the health system perpetuates power imbalances and does not value other entities in the provision of healthcare such as churches, community groups and culturally specific media outlet which have reach into their communities. There appeared to be an unwillingness by the health providers and non-Maori, non-Pacific clinicians to utilise resources already available, and to which patients and support people feel already engaged.

### (3.4) Collaboration

Collaboration in health care was discussed among participants as the willingness to work within a team, recognising the skills and expertise of others, and being willing to share responsibility for decisions about health care.

Participants in each of the focus groups discussed the critical nature of collaboration in the provision of high-quality care services for advanced COPD. Patients and carers reported on collaboration with health professionals. Clinicians described collaborations with colleagues and between their organisations. Health professionals mentioned examples of collaboration that enabled seamless and flexible care. These participants reported collaborative care at the clinician level being easier in certain settings. For example, in rural areas, clinicians were more likely to know each other, and these interpersonal relationships fostered streamlined care between organisations and individual practitioners.

*For paramedics it’s easy to transport, quicker to transport…workloads [are] heavy within a metropolitan area whereas rural clinicians often will speak to hospice and speak to Nurse Maude [community health care provider], look up Health Connect South [electronic medical record] and speak to the carer and the district nurse because the access is a little bit better. (Focus group 2, Health professional)*

Community-based clinicians also discussed spending much of their time communicating with other members of the health care team as a core aspect of their role.

Pacific participants described the importance of the medical profession engaging with them, to listen to their ideas of how the health system could be improved, and to learn what is already working.

*I think there’s a lot of work that needs to be done within the health system and within the supports given […], hopefully the government understands the worth of families, of churches, of our different community groups and to create that illness prevention strategy that focuses on working within our world view. That’s not just the physical, but also the spiritual, mental, financial, emotional, all those other wellbeing parts of our model. (Focus group 6, Health professional)*

Those with end-of-life COPD felt they had, as users of the system, valuable insights into how to improve their health care and wanted to share these insights with stakeholders who they perceived had the power to make actual change. All participants expressed the need for health care team members to validate and be respectful of everyone’s experience. Carers also described examples of high-quality care which occurred when health care teams prioritised the patient and family perspectives and goals.

*We went with elder care which were […] hospital then, 10 years ago. We went to […different] Hospital, he was assessed. We had everything put in place. Our daughter was online on the phone at that meeting hearing and speaking. They interviewed Bill on his own, they interviewed me, we got the whole nine yards and we had them come in. They put rails up, they put things in the bathroom. We got everything and it wasn’t because I pushed it. It was just there. (Focus group 1, support person)*

Patients and support people described experiences in which lack of collaboration led to the removal of choices, and created gaps in care delivery.

*The patient is not getting the care that they once used to get, so there’s less time spent with the patient and information like via doctor and nurses. I’m starting to see that there’s just a lot more pressure and stresses which is like a domino effect, it goes back onto the patients and their families, and you just see the frustration cause then it doesn’t go back out to sometimes the information gets missed out going back to the GP once the patient’s been discharged, and so there’s can be lost information where people just fall through the cracks. (Focus group 5, Māori support person)*

Patients also expressed frustration at the compartmentalisation of their different health problems and the separate care provided. They reported that the lack of holistic assessment by the health system was a barrier to high-quality care.

*I have other conditions as well as COPD. And I have extreme trouble getting them to understand I want someone to see to something else as well. That just seem to be so blinkered you know narrow tunnel vision, but they need to liaise more within their departments, I guess*.

*The different specialties?*

*Yes*.

*Yeah, it sounds like they basically just specialise in one thing and do not think outside the square to- Could do this or try that or something. (Focus group 3, Patient)*

This fragmentation of care occurred in the health system, and was also experienced in the social systems by participants. Government department staff did not appear to understand the severity of chronic lung disease and had unrealistic expectations of the affected person. Participants expressed frustration at the disconnection between health care professionals, organisations, and government entities, which led to worse outcome for them and their families. This disconnect led to feelings of frustration and helplessness, inadequate housing, lack of knowledge about entitlements or services that were available, and siloed care.

### (3.5) Community

Community was signposted by participants as people who shared a common interest and experienced a sense of belonging. Patient and carer participants particularly described several instances where a feeling of community directly improved their health and healthcare.

Many of the participants shared stories and encounters that other participants related to in a personal way. The discussions also focused on COPD and the different types of care that attendees had received, revealing a deep knowledge of the condition itself. Patients expressed the wish to help others in the same predicament through education and understanding.

*I’ve seen people and I go up and go, “You alright?” and I just lean with them because I know exactly what’s happening because I’ve got it. You see the signs. You’ll be sitting there and hear someone in the night I’ll go, “Oh, have you got an inhaler?” I’m really bad, I’d go like this, “What inhalers are you on?” (Focus group 5, Māori patient)*

For some participants, the sharing of their stories created a purpose for them and others in the groups. The bereaved carers showed strong support for each other in their grief, revealing gaps in the health care system.

*You go to WINZ [government organisation] and hang out there and go, why not. You go there because, holy […] man, what do I do? I’m in dire straits and I need some help, so that’s what you do. Yeah, and that is the sad thing, and it breaks my heart that you went through that and I wish I knew you in that way and I would’ve*

*I know you would have*

*I would have (Focus group 1, Support people)*

Numerous patients in the focus groups reported the positive impacts of the community-based “Better Breathing” classes. Patients enjoyed the challenge of exercise, being together in a supportive environment and gathering in a community setting. These support groups were described as combating isolation and loneliness. Participants described these groups as inclusive, and willing to cross boundaries.

*One of the two that came to your Better Breathing, they said if they hadn’t have seen you, they would have walked out because that’s that connection. They changed the seating. They didn’t like to be in a circle. They wanted to be more relaxed. In a circle, even though you’re all included, both of them felt that it was too intrusive until they got to know the group, which is fair enough. You don’t actually feel that you can say what you have to say, well, they didn’t feel that they could share what they had. (Focus group, Māori patient)*

Pacific participants described churches and craft groups as vital to their health care, where they felt a sense of belonging and engagement. Health care professionals also mentioned the vital part community played in provision of support for health issues, where they had witnessed better health care outcomes for those who had stronger connections with the community.

*It’s really striking that the people who manage the best at home, are not those people who have the best relationships with the clinicians but it’s those who have the best community supports, the best networks of informal carers. (Focus group 7, Hospital-based health professional)*

The absence of community between the health system and consumers of health was discussed by participants as creating an “us “and “them” culture. In the life of someone with COPD, participants encountered harsh interactions secondary to a lack of awareness of the illness. Participants described feeling helpless and hopeless in these situations, which they felt could be remedied by more public education. These encounters fostered a feeling of isolation and loneliness, which was sometimes able to be improved by health professionals but not necessarily in a co-ordinated way. Many patients with severe COPD in the focus groups had experienced exclusion and judgement from the community due to their condition.

*I cough because of phlegm and people just give me the most filthiest look…*

*Yeah*.

*Yeah*.

*…because I’ve walked too far or something’s happened and I’m out of breath and people just look. It’s alright. I’ve got a breathing problem. But still, you still get the evil eye. (Focus group 4, Patient)*

## (4) Discussion

Using Critical Theory and Actor-Network Theory, stakeholders’ perspectives and experiences identified five themes enabling quality care at end-of-life for people with COPD: compassion, competence, collaboration, community, and commitment. These aspects of quality care were understood as those that strengthen the network in which the stakeholders received and delivered care despite poor network structure and an absence of resources. These themes also identified how stakeholders explicitly and implicitly disrupted existing networks, and showed where the network for end-of-life COPD did not enable quality care. Participants expressed the importance of showing compassion, commitment, and the willingness to collaborate with their communities. There was increased trust in those health professionals who were proactive, with excellent communication skills and cultural competence. Healthcare networks engaged with people with COPD at the end-of-life have the opportunity to improve services by utilising these findings.

Healthcare networks have been promoted as “making wicked problems governable” [20]. Networks that have successfully united relevant stakeholders to a common goal and negotiated joint priorities have improved health outcomes [21,22]. Studies have evaluated healthcare networks with an emphasis on identifying the components which most influence the effectiveness of the network. Collaboration between stakeholders, similar to our findings, has been mentioned as a critical factor for success [20,22]. Collaborative practices such as formalising arrangements between stakeholders, either through contracts or memoranda of understanding, are proven to strengthen relationships [22,23].

The presence of a hierarchy within networks disrupts collaboration and impacts the sense of community among individual and organisational stakeholders [24]. A network that has multiple actors being aware of others and their well-being creates a sense of compassion towards colleagues [21]. When this phenomenon is absent, lower quality interactions among stakeholders and resource allocation weakens the network and negatively affects health outcomes. Leadership of health networks that promotes trust and values interactions between stakeholders is vital to an effective network [20,21,25]. The commitment of management and leaders to effect change management and communicate change to individual stakeholders made positive improvements to healthcare outcomes [20]. Healthcare professionals tended to be motivated to participate in healthcare networks as these entities were viewed as able to make a genuine difference to the quality of care, alongside like-minded colleagues [22].

These motivations parallel our research finding of commitment being vital to the strengthening of healthcare networks. Ko Awatea is a health network that was established in 2011 to improve health services in Counties Manukau, Aotearoa New Zealand through fostering a culture of professional development, information transfer, and collaborative innovation. The healthcare network included an education hub for students, staff and visitors to empower health professionals and the community, which reduced adverse medical outcomes [26]. The concept of ‘shared learning’ positively strengthens networks by fostering connections, upskilling stakeholders (competence) and contributing to the creation of community [20]. Similar to our study, the Ko Awatea team discovered collaboration, commitment to a shared vision, prioritisation of competence and a sense of community all played a role in the improvement of health care [27], although the evaluation did not include patients.

A study undertaken between 2005 and 2009 in the National Health Service in the UK compared healthcare networks across a number of sectors, including end-of-life care among older adults [20]. This qualitative study evaluated the contributors to network performance, both positively and negatively, by interviewing stakeholders, excluding patients. The network was multi-disciplinary and involved several cross-agency relationships, similar to our severe COPD healthcare network. A variety of disruptions were identified mainly due to a lack of collaboration and commitment between organisations, impeding healthcare outcomes [20].

### (4.1) Applications for practice and research

The incorporation of the core elements of commitment, compassion, competence, collaboration, and community strengthen a healthcare network and is associated with high quality care. Each healthcare network will already involve organisations and individuals with strengths in different areas and these positive traits could be intentionally maximised without incurring extra cost. Other recommendations include building organisational cultures that allow compassion and commitment to flourish, with intentional efforts to flatten hierarchies. Engagement with relevant community-based individuals and services, valuing their expertise, engenders synergies in provision of care. Training and education in medical knowledge, parallel to cultural competencies and communication skills are vital for high quality care. Projects funded by government or healthcare systems which prioritise these features in an authentic way may be associated with higher-quality care across settings and conditions.

### (4.2) Strengths and limitations

This research project was conducted in a large district of Aotearoa New Zealand, and included patients, support people and health professionals. A wide variety of people were involved in the focus groups, which were facilitated by skilled and culturally concordant researchers. The analysis was performed using the frameworks of Critical Theory and Actor-Network theory which allowed prioritisation of the participants as experts in the care of people with severe COPD. A research advisory group was consulted throughout the design, implementation and interpretation of the research project to identify researcher bias and enable a wider lens on the study conduct. The generalisability of these study results to other health systems and medical conditions is unknown. Aotearoa New Zealand has a unique culture and health system which may influence interpretation of the results. Although the participants were diverse in age and ethnicity, the team did not include more marginalised populations such as people without homes or migrant people.

### (4.3) Conclusions

Prioritising the voices of those with severe COPD, their support people and health professionals about their experiences of end-of-life care led to the identification of critical features of high-quality care.

## Data accessibility statement

Participants only consented for the data to be utilised for this research project. The data is not available outside the research team.
